# Arachidonic acid-dependent gene regulation during preadipocyte differentiation controls adipocyte potential[S]

**DOI:** 10.1194/jlr.M049551

**Published:** 2014-12

**Authors:** Evanthia Nikolopoulou, Georgia Papacleovoulou, Frederic Jean-Alphonse, Giulia Grimaldi, Malcolm G. Parker, Aylin C. Hanyaloglu, Mark Christian

**Affiliations:** * Institute of Reproductive and Developmental Biology, Department of Surgery and Cancer, Faculty of Medicine, Imperial College London, London, UK; † Division of Women’s Health, King’s College London, London, UK; § Division of Metabolic and Vascular Health, Warwick Medical School, University of Warwick, Coventry, UK

**Keywords:** adipogenesis, prostaglandins, cell signaling, metabolism

## Abstract

Arachidonic acid (AA) is a major PUFA that has been implicated in the regulation of adipogenesis. We examined the effect of a short exposure to AA at different stages of 3T3-L1 adipocyte differentiation. AA caused the upregulation of fatty acid binding protein 4 (FABP4/aP2) following 24 h of differentiation. This was mediated by the prostaglandin F_2α_ (PGF_2α_), as inhibition of cyclooxygenases or PGF_2α_ receptor signaling counteracted the AA-mediated aP2 induction. In addition, calcium, protein kinase C, and ERK are all key elements of the pathway through which AA induces the expression of aP2. We also show that treatment with AA during the first 24 h of differentiation upregulates the expression of the transcription factor Fos-related antigen 1 (Fra-1) via the same pathway. Finally, treatment with AA for 24 h at the beginning of the adipocyte differentiation is sufficient to inhibit the late stages of adipogenesis through a Fra-1-dependent pathway, as Fra-1 knockdown rescued adipogenesis. Our data show that AA is able to program the differentiation potential of preadipocytes by regulating gene expression at the early stages of adipogenesis.

Adipocytes are the primary site for fat storage with 90% of the adipocytes’ cytoplasm containing a large lipid droplet (1). Apart from their role in fat storage, adipocytes are the major players in handling the lipid distribution across the body. Adipocytes can synthesize and take up lipids by sensing insulin, and can also hydrolyze and secrete lipids in response to β-adrenergic receptor signaling. In addition, adipose tissue is a well-characterized endocrine organ that secretes hormones and regulates lipid homeostasis across the body (2). Adipogenesis, the process of adipocyte differentiation, was initially thought not to proceed beyond the formation of adipose tissue, i.e., after the prenatal and early postnatal period. However, data have shown that new adipocytes can be formed during adulthood (3).

Dietary fat has been correlated with increased adipocyte number (hyperplasia) and size (hypertrophy) (4). However, it is now apparent that this is a simplistic view, as fats have different effects on adiposity depending on their nature and properties. The role of arachidonic acid (AA), a major n-6 PUFA, on obesity is controversial from studies using both in vivo and in vitro models (5–10). However, it has been demonstrated that the protein/carbohydrate ratio in the diet can determine the role of AA on adiposity (11). AA can be metabolized to a diverse group of metabolites, depending on the presence and activity of catalyzing enzymes. Prostaglandins (PGs), the products of AA generated by cyclooxygenases (COXs), have opposing effects on adipogenesis, as prostacyclin (PGI_2_) and 15-deoxy-Δ^12,14^ prostaglandin J_2_ (PGJ_2_) promote (12–15) and prostaglandin E_2_ (PGE_2_) and prostaglandin F_2α_ (PGF_2α_) inhibit adipocyte differentiation (16–18).

Due to the important role of lipids in adipocytes, it is not surprising that fatty acid protein 4 (FABP4 or aP2) constitutes 5% of the total protein of an adipocyte (19). aP2 is a lipid chaperone binding hydrophobic ligands that facilitates the transport of lipids to specific compartments in the cell (20), interacts with various players of the regulatory mechanisms of adipocyte function (21), and translocates to the nucleus affecting gene expression (22). However, the function of aP2 is still poorly understood, as aP2 knockout mice show insulin sensitivity in the context of both dietary and genetic obesity, but not in lean mice (23). The expression of aP2 is induced during adipocyte differentiation, as it is transcriptionally controlled by the master regulator of adipogenesis, PPARγ (24). PPARγ is activated by natural (fatty acids) or synthetic agonists (such as thiazolidinediones), which also lead to aP2 upregulation (13–15, 25, 26).

Activator-protein 1 (AP-1) transcription factors have been implicated in both adipogenesis (27) and regulation of aP2 expression (28). The AP-1 family consists of the Fos [Fos-related antigen (Fra)-1, c-Fos, FosB, and Fra-2] and Jun proteins (c-Jun, JunB, and JunD), which can heterodimerize (29). Fra-1 is an AP-1 factor that is of particular interest in adipogenesis, as Fra-1 transgenic mice have severe lipodystrophy with Fra-1 inhibiting CCAAT/enhancer binding protein α (C/EBPα) expression (30). It is induced early in the adipocyte differentiation program (31) and its expression is enhanced by PPARγ agonists (32), fatty acids (33), mitogens (34), and insulin (35). Little is known about Fra-1 target genes in preadipocytes and its mode of action, as it could be both an activator and a repressor (36, 37).

As AA is a major n-6 PUFA and a significant signaling molecule abundant in the Western diet, it is important to understand how it regulates adiposity through the process of adipocyte differentiation. Hence, the current study aimed to elucidate the effects of AA at the early stages of adipogenesis and delineate the events initiated by AA to program the differentiation ability of preadipocytes. We demonstrate that AA, after 24 h of treatment, is metabolized by COXs to generate PGF_2α_, which binds to its membrane G protein-coupled receptor (GPCR), the FP receptor, and in turn induces the calcium/protein kinase C (PKC)/ERK signaling pathway that causes the upregulation of aP2 and Fra-1 expression. We also show that the inhibition of terminal differentiation by AA present at the early stages of adipogenesis is Fra-1-dependent, as Fra-1 knockdown could rescue the inhibitory effect of AA on differentiation.

## MATERIALS AND METHODS

### Reagents

Carbaprostacyclin (cPGI_2_), AL 8810, baicalein, and 17-octadecynoic acid (17-ODYA) were purchased from Cayman Chemical. 15-Deoxy-Δ^12,14^ PGJ_2_, PGE_2_, PGF_2α_, SC-560, SC-236, and indomethacin were all purchased from Sigma-Aldrich. PD98059, U0126, and GF109203X were obtained from Enzo Life Sciences and BAPTA-AM from Tocris Bioscience.

### Cell culture

3T3-L1 cells were maintained in subconfluent culture in DMEM containing 4.5 g/l glucose and L-glutamine (Invitrogen) supplemented with 10% newborn calf serum (Invitrogen) and antibiotics (Sigma-Aldrich) at 37°C in 5% CO_2_. For the short-term experiments, 48 h after reaching confluence (day 0), cells were treated with a differentiation cocktail (MDI) of 0.5 mM methylisobutylxanthine (Sigma-Aldrich), 250 nM dexamethasone (Sigma-Aldrich), and 170 nM insulin (Sigma-Aldrich) in the presence of AA or BSA (vehicle) for 24 h. For the long-term experiments, cells were cultured and treated the same as the short-term experiments until 24 h, when the AA or BSA was withdrawn. The MDI cocktail was then kept for an additional 24 h (day 2), when it was replaced by culture medium containing insulin (170 nM) until the end of the differentiation program (day 10). The medium was replenished every 2 days. For ERK experiments, 2 day postconfluent cells were serum-starved overnight and then assayed as described. For the pretreatment experiments, 2 day postconfluent cells were preincubated with the respective inhibitors for 30 min (see the Results section) followed by AA or BSA treatments for 24 h in the presence of MDI.

For siRNA-mediated FP receptor (siFP) and Fra-1 (siFra-1) knockdown, 80–90% confluent 3T3-L1 cells were transfected with siRNA (ON-TARGETplus SMARTpool, mouse FOSL1 or mouse PTGFR, Dharmacon) or scrambled sequences (siControl) with Dharmafect 3 (Dharmacon), according to the manufacturer’s instructions. The cells were treated on the third day after transfection and were either harvested after 24 h or retransfected and harvested at the end of the differentiation program.

### Preparation of albumin-bound fatty acid

Stock solution of AA (10 mM) (Cayman Chemical) was prepared by diluting the FFA in ethanol and precipitating it with the addition of NaOH (final concentration of 0.25 M). The precipitated sodium salt was then evaporated under nitrogen gas, reconstituted with 0.9% (w/v) NaCl, and stirred at room temperature for 10 min with fatty acid-free BSA (Sigma-Aldrich) in 0.15 M NaCl. The solution was stored in multiple aliquots at −20°C and protected from light in tubes evacuated under nitrogen gas. Control solution containing fatty acid-free BSA was similarly prepared. The FFA/BSA molar ratio was 4:1 (38). All AA treatments contained 0.22 mM α-tocopherol (Sigma-Aldrich) to prevent lipid peroxidation (39).

### Western blotting

Cells were lysed in ice-cold lysis buffer containing 1% Triton-X, 50 mM Tris-HCl (pH 7.4), 150 mM NaCl, 0.5 mM EDTA, 1 mM PMSF (Sigma-Aldrich), 1× protease inhibitor cocktail (Roche), and 1× phosphatase inhibitor cocktail (Sigma-Aldrich). Protein concentration was determined using the BCA^TM^ protein assay kit (Pierce). Equal amounts of protein were resolved by SDS-PAGE and electro-transferred onto a polyvinyl difluoride membrane (Millipore). Blots were blocked in 5% nonfat dry milk or 5% BSA in 1× TBS-T (TBS with 0.1% TWEEN 20). Primary antibody was applied in the same buffer at a dilution of 1:1,000. Primary antibodies were purchased either from Cell Signaling Technology [phospho-p44/42 (9101) and total ERK (9102)], Millipore [GAPDH (MAB374)], or Santa Cruz Biotechnology [Fra-1 (sc-605)]. Horseradish peroxidase-conjugated secondary antibodies (Invitrogen) were applied in a dilution of 1:5,000. Finally, the signal was detected using enhanced chemiluminescence substrates (Pierce) following the manufacturer’s instructions and the light emission was captured and visualized by exposing the blot to film. Densitometry was carried out by analyzing Western blot images with ImageJ software.

### Quantitative RT-PCR

Total RNA was isolated from cells using TRI Reagent (Sigma-Aldrich) and reverse transcribed to cDNA using M-MLV reverse transcriptase (Invitrogen), following the manufacturer’s in­structions. Real-time RT PCR was performed on a 7900HT Fast real-time PCR system (Applied Biosystems) using SYBR® Green JumpStart™ Taq ReadyMix™ (Sigma-Aldrich). The primers used for real-time RT-PCR were: aP2, forward (Fw) 5′-ACACCGAGATTTCC­TT­CAAACTG-3′ and reverse (Rv) 5′-CCATCTAGGGTTATGATGC­TCTTCA-3′ PPARγ2, Fw 5′-TGGG­TGAAACTCTGG­GA­GA­TTC-3′ and Rv 5′-AGAGGTCCACAGAG­CTGATTCC-3′ c/EBPα, Fw 5′-TGGAGTTGACCAGTGACA­ATGAC-3′ and Rv 5′-CAGTT­CA­CG­GCTCAGCTGTTC-3′ FAS, Fw 5′-TGCGGAAA­CTTCA­GG­AAATGT-3′and Rv 5′-AGAGACGTGTCACTCCTGGACTT-3′ Fra-1, Fw 5′-CGCAAGCTCAGGCA­CAGA-3′ and Rv 5′-AATGA­GGCTGCACCATCCA-3′ FP receptor, Fw 5′-TTC­A­GCT­CCT­GGCCATAATGT-3′ and Rv 5′-AAAAAGTGTCGTTTCACAGG­T­CACT-3′ and L19, Fw 5′-GGAAA­AAGA­AGG­TCT­GG­TTG­GA-3′ and Rv 5′-TGATCTGCTGACGGGAGTTG-3′.

### Oil Red O staining

3T3-L1 cells were stained with Oil Red O (Sigma-Aldrich). The culture medium was removed and the cells were washed with PBS. Cells were then fixed with 4% paraformaldehyde in PBS for 15 min at room temperature. The fixative was discarded and the cells were washed with PBS. Oil Red O (0.15%. w/v) in 60% isopropanol was applied for 1 h at room temperature. The cells were then rinsed with 60% isopropanol and PBS. Stained lipid droplets were visualized under light microscopy.

### Calcium mobilization assay

Two day postconfluent 3T3-L1 cells were assayed for calcium mobilization using the Fluo-4 Direct™ calcium assay kit (Invitrogen). On the day of the assay, the dye was prepared according to the manufacturer’s instructions. Briefly, 2× calcium sensitive Fluo-4 Direct buffer was mixed with the same volume of differentiation cocktail (MDI) and cells were incubated for 30 min in an incubator (5% CO_2_ at 37°C). The cells were then incubated for an additional 30 min at room temperature. Plates were then placed for live cell imaging on an SP5 confocal microscope (Leica) using the LAS-AF program. Dishes were left for 1 min to gain a basal value before being applied with AA (10 μM) for 10**–**15 min. The data were exported from the program as values based on the fluorescence emitted during the experiment, with the basal average intensity from the first 1 min subtracted.

### Statistical analysis

Relative mRNA levels of target genes were normalized to L19 expression and the effects of different treatments were expressed as fold changes to the equivalent control treatments. Results were analyzed using the Student’s *t*-test. *P* values lower than 0.05 were considered statistically significant.

## RESULTS

### Short-term treatment with AA induces aP2 expression in preadipocytes

**Fig. 1.**
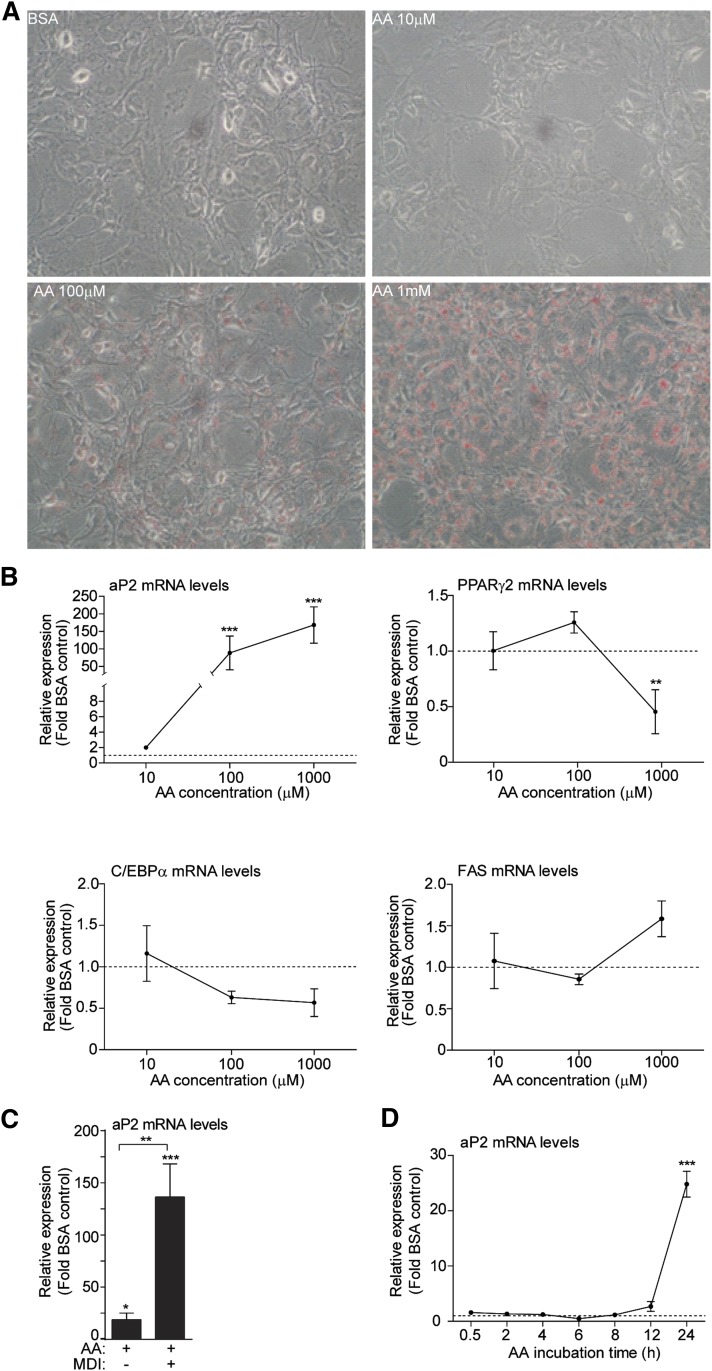
AA induces the expression of aP2 after 24 h of treatment in 3T3-L1 cells. A: Oil Red O stain­ing of 2 day postconfluent 3T3-L1 cells (day 0) upon AA treatment (10 μM, 100 μM, and 1 mM) or fatty acid-free BSA (vehicle for AA) for 24 h. Cells were captured under a light microscope using 20× magnification. B: 3T3-L1 cells (day 0) were incubated for 24 h with BSA vehicle (set as 1, dashed line) or AA (10 μM, 100 μM, and 1 mM) in the presence of MDI. Total RNA was harvested and RT-PCR was performed. Mean values are shown of n = 3 and error bars represent ±SEM. Statistical significance was determined by a Student’s *t*-test (two-tailed). **P < 0.01, ***P < 0.001. C: 3T3-L1 cells (day 0) were treated with AA in the presence or absence of MDI for 24 h. A Student’s *t*-test was performed for the BSA control and AA-treated for each condition and between AA-treated in different conditions. Error bars represent ±SEM. *P < 0.05, **P < 0.01, and ***P < 0.001. D: 3T3-L1 cells (day 0) were treated with 100 μM AA in the presence of MDI and total RNA was prepared at the indicated time points. Data are presented as mean ± SEM based on triplicate determinations. A Student’s *t*-test was performed for the 24 h time point between the vehicle-treated and the AA-treated. ***P < 0.001.

To test whether AA affects gene expression at the early stages of differentiation, 3T3-L1 cells were treated with increasing doses of AA (10 μM, 100 μM, and 1 mM) for the first 24 h of differentiation in the presence of standard differentiation cocktail (MDI). These doses were selected because fatty acids can be found in the plasma of fed or fasted mice between a range of 0.1 to 1.2 mM and have been used in prior in vitro studies (33, 40). Initially, we observed that lipid droplet formation was increased proportionally with the AA concentration (**Fig. 1A**). To examine whether AA promotes the early terminal differentiation of preadipocytes, the expression of late gene markers of differentiation was assessed, such as aP2, PPARγ2, C/EBPα, and FAS, following 24 h of treatment with AA. aP2 was the only late differentiation gene marker that was upregulated by AA in a dose-dependent manner (Fig. 1B). A significant, but not as dramatic, increase in aP2 levels was also observed following 24 h treatment with AA in the absence of MDI (Fig. 1C). To examine whether the effect of AA on aP2 expression occurs earlier than 24 h, time-course experiments were performed with 100 μM AA in the presence of MDI. We observed that the aP2 mRNA expression was significantly upregulated only after 24 h of AA treatment, but not at earlier time points (Fig. 1D). Our results suggest that the upregulation of aP2 expression by AA was a gene-specific effect rather than an effect on the differentiation program.

### PGF_2α_ mediates the effect of AA on aP2 expression

**Fig. 2.**
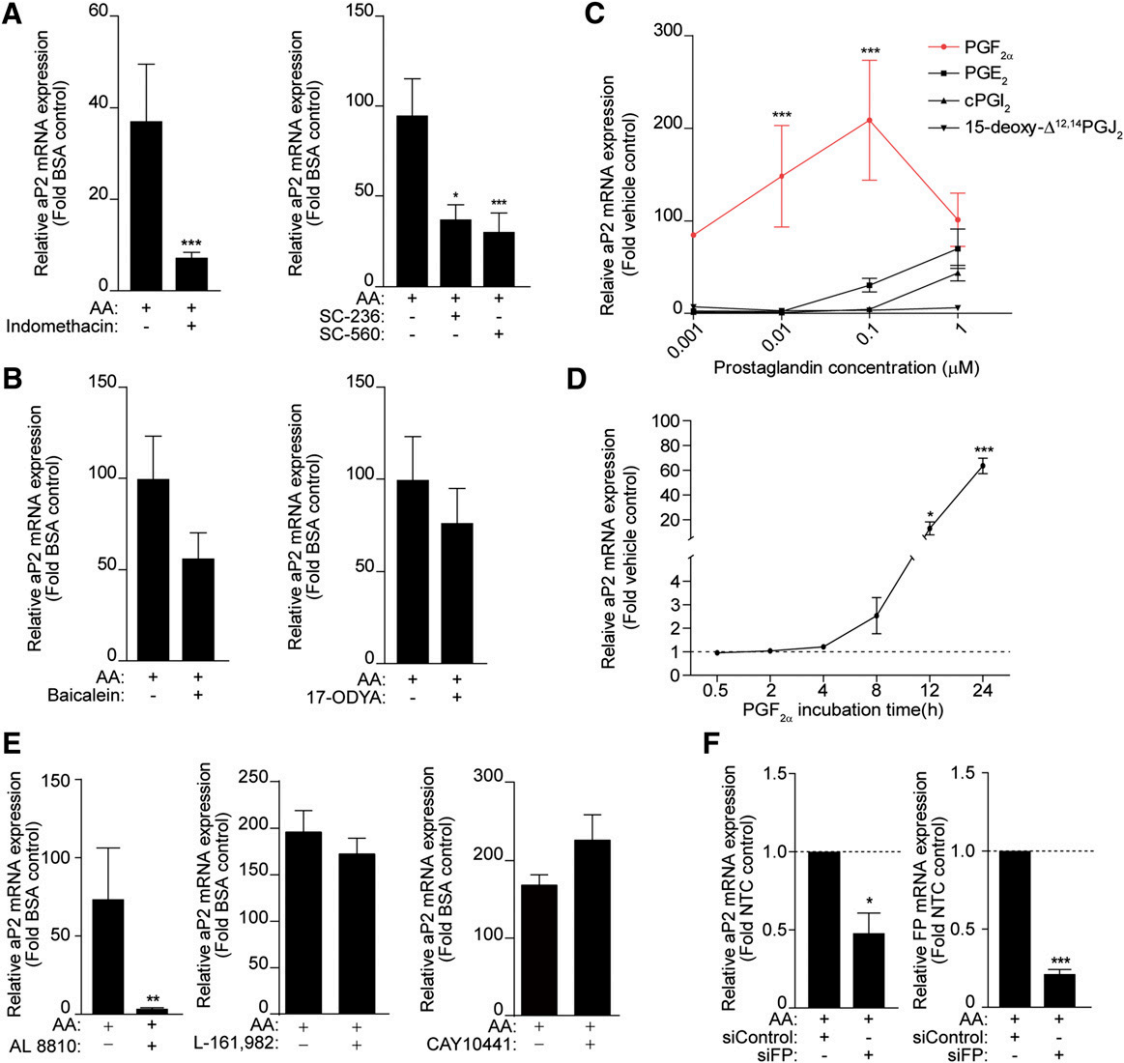
PGF_2α_ mediates the effect of AA on aP2 expression in 3T3-L1 cells. 3T3-L1 cells (day 0) were pretreated with indomethacin (10 μM), SC-236 (10 μM), and SC-560 (10 μM) (A) and baicalein (10 μM) or 17-ODYA (10 μM) (B) for 30 min prior to AA (100 μM) treatment for 24 h in the presence of MDI. Total RNA was isolated and RT-PCR was performed. C: 3T3-L1 cells (day 0) were treated with 1 nM, 10 nM, 100 nM, or 1 μM of PGF_2α_, PGE_2_, cPGI_2_, and 15-deoxy-Δ^12,14^ PGJ_2_ for 24 h in the presence of MDI. The red line is the PGF_2α_ treatment. D: 3T3-L1 cells (day 0) were treated with PGF_2α_ (10 nM) in the presence of MDI for 24 h. E: 3T3-L1 cells (day 0) were pretreated with AL 8810 (10 μM), L-161,982 (10 μM), or CAY10441 (10 μM) for 30 min prior to 100 μM AA treatment for 24 h. F: Eighty percent confluent 3T3-L1 cells were transfected with a nontargeting control siRNA (siControl) or siRNA targeting the FP receptor (siFP). On the third day after transfection, cells were treated with 100 μM AA for 24 h in the presence of MDI. RT-PCR was conducted for aP2 mRNA and FP receptor transcripts. Data are presented as mean ± SEM of n = 3. *P < 0.05, **P < 0.01, ***P < 0.001.

AA is a substrate of enzymes in the eicosanoid pathway [COXs, lipoxygenases (LOXs), and P450 epoxygenases] producing a variety of metabolites. To examine whether these derivatives of AA have a role in the regulation of aP2 expression, 3T3-L1 cells were pretreated with either indomethacin (a general COX inhibitor), a selective COX-2 (SC-236) and a COX-1 inhibitor (SC-560), baicalein (a 12/15 LOX inhibitor), or 17-ODYA (a cytochrome P450 epoxygenase inhibitor). Indomethacin and the selective COX inhibitors significantly blocked the AA-dependent induction of aP2 mRNA levels (**Fig. 2A**) and the expression of both COX-1 and -2 was upregulated by AA in a dose-dependent manner (supplementary Fig. I). However, the effect of AA was not blocked by the LOX or epoxygenase inhibitors (Fig. 2B), indicating that PGs mediate the effect of AA on aP2 expression.

To identify which PGs mediate the increase in aP2 expression by AA, a dose response experiment was carried out treating 3T3-L1 cells with either carbaprostacyclin (cPGI_2_; an analog of PGI_2_), PGF_2α_, PGE_2_, or 15-deoxy-Δ^12,14^ PGJ_2_ for 24 h in the presence of MDI. PGF_2α_ had a similar effect to AA on aP2 expression (Fig. 2C, red line), where at the lowest concentration (1 nM) tested, it was able to upregulate aP2 mRNA levels almost 100-fold. PGE_2_ had a promoting effect on aP2 expression at 100 nM (30-fold) and cPGI_2_ at 1 μM (40-fold) (Fig. 2C). However, 15-deoxy-Δ^12,14^ PGJ_2_, which is a direct PPARγ ligand (13–15), did not have a significant effect at any of the doses tested (Fig. 2C). To further examine whether PGF_2α_ mimics the effect of AA, a time-course experiment was performed treating 3T3-L1 cells with PGF_2α_ (10 nM) over the course of 24 h. PGF_2α_ significantly induced aP2 mRNA expression after 12 h and 24 h of treatment (Fig. 2D), suggesting that it mediates the AA-dependent upregulation of aP2 expression. As both PGE_2_ and PGI_2_ could upregulate aP2 expression (Fig. 2C), preadipocytes were pretreated with an EP4 selective antagonist (L-161,982, 10 μM) or a prostacyclin receptor (IP)-selective antagonist (CAY10441, 10 μM) (Fig. 2E) prior to AA treatment. These antagonists were unable to block the effect of AA on aP2 expression, suggesting that these PGs are not mediating the effects of AA (Fig. 2E). The weaker effects of PGE_2_ and cPGI_2_ on aP2 expression (Fig. 2C) could be via the known ability of these PGs to bind weakly to the FP receptor (*K_i_* of 100 nM and 1,200 nM, respectively) (41).

To further confirm that PGF_2α_ is the metabolite mediating aP2 expression by AA, we blocked the signaling initiated by PGF_2α_ binding to its membrane GPCR receptor, the FP receptor. 3T3-L1 cells were either pretreated with the FP receptor antagonist (AL 8810, 10 μM) or depleted of the FP receptor by a targeting siRNA (siFP) prior to AA treatment (Fig. 2E, F). The induction of aP2 expression by AA was significantly blocked in the presence of AL 8810 (Fig. 2E) and reduced by 50% in the presence of siFP when the FP receptor mRNA levels were reduced around 80% (Fig. 2F). Overall, these results confirm that PGF_2α_, via its membrane FP receptor, mediates AA-induced increase in aP2 expression early in differentiation.

### PGF_2α_ signaling pathway regulates aP2 expression

**Fig. 3.**
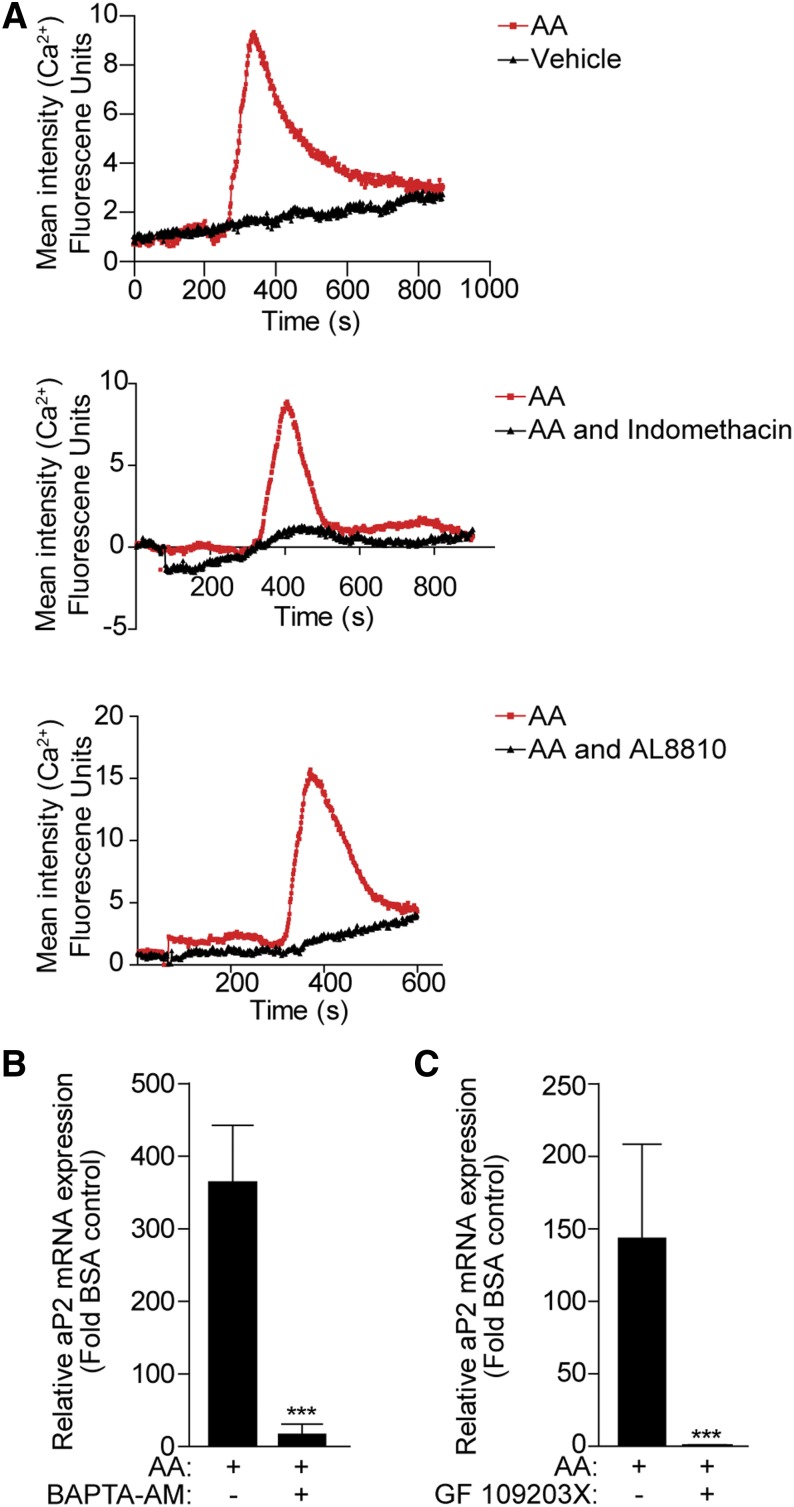
The calcium and PKC signaling pathway mediates the effect of AA on aP2 expression. A: 3T3-L1 cells (day 0) were loaded with calcium-sensitive Fluo-4 before stimulation with 10 μM AA (red line) or BSA-vehicle (black line), and calcium mobilization was determined by live cell imaging for 10–15 min (top panel). Cells were pretreated with indomethacin (10 μM) (middle panel) or AL 8810 (10 μM) (lower panel) for 30 min before stimulation with AA (10 μM). A representative experiment from three independent experiments is shown. B, C: 3T3-L1 cells (day 0) were pretreated with BAPTA-AM (30 μM) or GF109203X (10 μM), as indicated, for 30 min prior to 100 μM AA treatment for 24 h. Total RNA was extracted and subjected to RT-PCR for aP2 mRNA transcript. Data are presented as mean ± SEM of n = 3. ***P < 0.001.

The FP receptor is coupled to the Gαq/11 family of G proteins, which activates the phospholipase C/phosphoinositide signaling pathway leading to mobilization of calcium from intracellular stores (42). Hence, calcium mobilization studies were performed to determine whether AA activates a calcium signaling pathway via the FP receptor. Two days postconfluent 3T3-L1 cells were loaded with the calcium dye Fluo-4 for live cell imaging. Addition of 10 μM AA induced a rapid and robust increase in intracellular calcium (**Fig. 3A**, upper panel). This effect was PG-mediated, as pretreatment with indomethacin completely blocked the mobilization of calcium stores (Fig. 3A, middle panel). Finally, addition of the FP receptor antagonist (AL 8810, 10 μM) was able to block the effect of AA on the intracellular calcium levels, confirming that the effect of AA on calcium increase was FP-mediated (Fig. 3A, lower panel).

To address whether calcium is required for the AA-induced expression of aP2 expression, 3T3-L1 cells were pretreated with a membrane-permeate calcium chelator (BAPTA-AM, 30 μM) prior to AA. BAPTA-AM significantly blocked the upregulation of aP2 expression by AA (Fig. 3B). Finally, it is known that calcium ions, in conjunction with diacylglycerol, activate PKC (42). Hence, in order to examine whether PKC is involved in the regulation of aP2 expression by AA, a PKC inhibitor (GF109203X, 10 μM) was added to 3T3-L1 cells prior to treatment with AA for 24 h. The induction of aP2 expression was completely blocked by preincubation with the PKC inhibitor, suggesting that AA activates a calcium/PKC pathway via the FP receptor that activates aP2 gene expression (Fig. 3C).

**Fig. 4.**
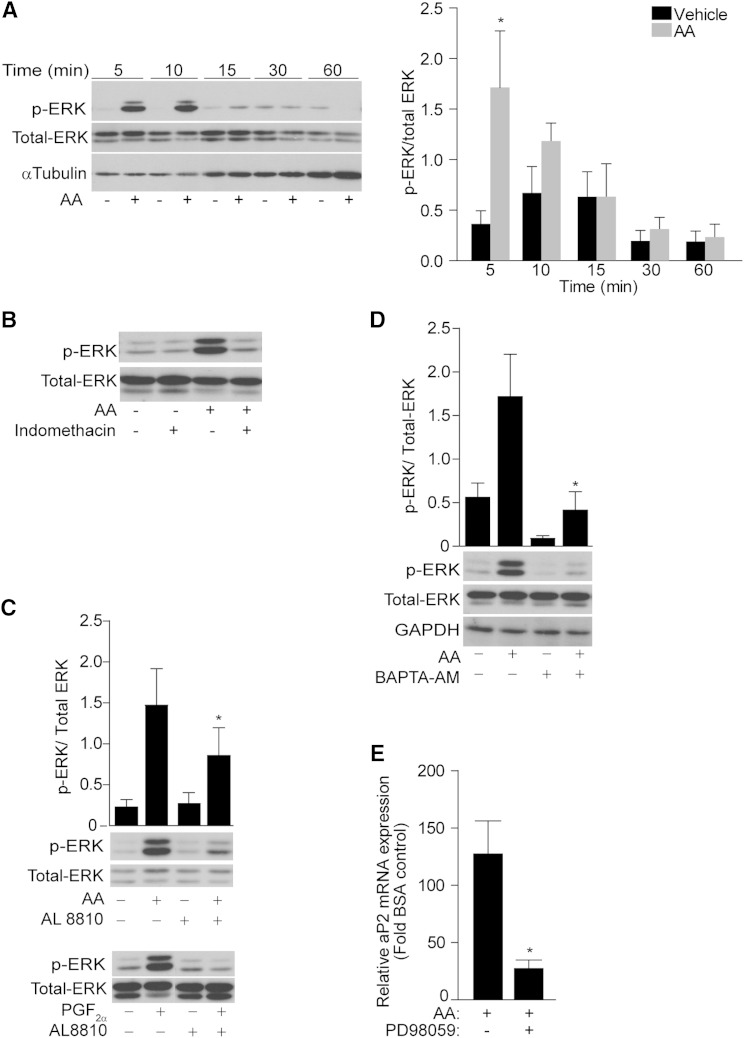
ERK kinase mediates the effect of AA on aP2 expression. A: 3T3-L1 cells (day 0) were serum starved overnight and treated with 10 μM AA for the indicated time points. Western blotting was performed using antibodies against phosphor-ERK, total ERK, and GAPDH. The graph shows the densitometric analysis. B: Western blot of phosphor-ERK and total ERK of serum-starved cells pretreated with indomethacin (1 μM) for 30 min prior to AA (10 μM) stimulation for 5 min. C: As above, but cells were pretreated with AL 8810 (10 μM) prior to AA (10 μM) (top panel) and PGF_2α_ (10 nM) (bottom panel), or with BAPTA-AM (10 μM) prior to AA (10 μM) (D). E: Cells were pretreated with PD98059 (10 μM) for 30 min prior to AA (100 μM) treatment for 24 h in the presence of MDI. aP2 mRNA levels are presented as fold change of the vehicle control from n = 3. Error bars represent ±SEM. *P < 0.05. Densitometry was performed of three independent blots normalizing the relative intensity of phosphor-ERK to the corresponding total ERK. *P < 0.05.

As both AA and PGF_2α_ are known to activate ERK signaling in differentiating cells (43, 44), we assessed whether AA-induced aP2 expression requires ERK activation. We first determined the temporal profile of ERK activation by AA in preadipocytes. A time-course experiment (0–60 min) was performed treating 3T3-L1 cells with AA (10 μM). AA activated ERK following 5 min of treatment, which was decreased to basal levels following 15 min of AA treatment (**Fig. 4A**).

As shown in Fig. 2, the effect of AA on aP2 expression was blocked by COX inhibitors, indicating a role for PG production. To address whether AA-induced ERK activation is PG-mediated, 3T3-L1 cells were pretreated with indomethacin for 30 min and then stimulated for 5 min with AA. Indomethacin completely blocked ERK activation by AA, indicating that this signaling pathway is PG dependent (Fig. 4B). To investigate whether PGF_2α_ mediates the AA-dependent activation of ERK, 3T3-L1 cells were pretreated with the FP antagonist, AL 8810 (10 μM), for 30 min prior to the addition of AA (10 μM, 5 min) (Fig. 4C, upper panel). Cells were also treated with PGF_2α_ (10 nM) in the presence or absence of AL 8810 (10 μM) as a positive control (Fig. 4C, lower panel). AL 8810 could significantly block the phosphorylation of ERK by AA, indicating that PGF_2α_ is the PG-activating ERK when cells are challenged with AA (Fig. 4C). It should be noted that the PGF_2α_ concentration able to activate ERK was 1,000 times lower than the concentration of AA able to achieve the same activation. The high concentration of AA required to activate ERK indicates that a metabolite of AA, rather than AA itself, mediates this effect. In addition, BAPTA-AM significantly blocked AA-mediated ERK activation, indicating that calcium signaling is required for AA-mediated ERK activation (Fig. 4D).

Finally, preadipocytes were pretreated with a mitogen-activated protein kinase kinase (MEK) inhibitor (PD98059, 10 μM) before the exposure to AA for 24 h in the presence of MDI. PD98059 significantly blocked the AA-mediated upregulation of aP2 mRNA expression (Fig. 4E), indicating that ERK signaling activated by AA is required for the regulation of aP2 expression.

### AA upregulates Fra-1 expression through PGF_2α_ signaling

**Fig. 5.**
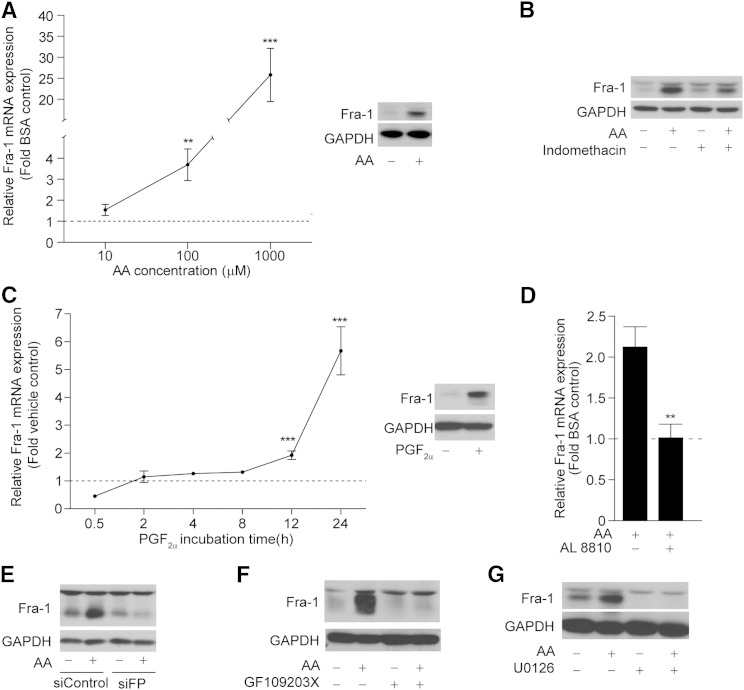
AA induces the expression of Fra-1 after 24 h of treatment in 3T3-L1 cells. A: 3T3-L1 cells (day 0) were treated with increasing doses of AA (10 μM, 100 μM, or 1 mM) for 24 h in the presence of MDI. Total RNA (left panel) or total cell lysates (right panel, 100 μM) were isolated and subjected to RT-PCR or Western blotting, respectively. B: Western blot of Fra-1 in cells pretreated with indomethacin (10 μM) for 30 min prior to AA (100 μM) treatment for 24 h. C: Cells were treated for 24 h with PGF_2α_ (10 nM) and total RNA (left panel) or whole cell lysates (right panel, 24 h) were harvested at the indicated time points and subjected to RT-PCR or Western blotting, respectively. D: Cells were pretreated with AL 8810 (10 μM) for 30 min prior to AA (100 μM) for 24 h. Total RNA was extracted and subjected to RT-PCR for Fra-1 mRNA transcript. E: 80% confluent 3T3-L1 cells were transfected with siControl or siFP. On the third day after transfection, cells were treated with 100 μM AA for 24 h in the presence of MDI. Western blot was performed with antibodies against Fra-1 and GAPDH. F, G: Cells were pretreated with GF109203X (10 μM) or U0126 (10 μM) for 30 min prior to AA (100 μM) stimulation for 24 h. Western blot was performed using antibodies against Fra-1 and GAPDH. Fra-1 mRNA levels are presented as fold change of the vehicle control from n = 3. Error bars represent ±SEM. **P < 0.01, ***P < 0.001.

The ERK pathway has been reported to regulate Fra-1 expression at both a transcriptional and posttranslational level (45). This observation, along with the finding that Fra-1 is expressed early in adipocyte differentiation (31) and is induced by fatty acids (33), led us to assess whether AA regulates Fra-1 expression. 3T3-L1 cells were treated with increasing doses of AA in the presence of MDI and harvested after 24 h of treatment. Fra-1 mRNA levels increased proportionally with increasing doses of AA. Protein levels also increased after 24 h of AA treatment (100 μM) (**Fig. 5A**). To confirm that Fra-1 expression is regulated by AA via the same pathway as aP2, 3T3-L1 cells were pretreated with indomethacin before treatment with AA, and Fra-1 protein levels were assessed. Not only was Fra-1 upregulation by AA blocked by the general COX inhibitor, but PGF_2α_ was also able to mimic the effect of AA on Fra-1 mRNA and protein expression (Fig. 5B, C). To determine whether the effect of AA on Fra-1 expression is FP receptor-mediated, 3T3-L1 cells were either pretreated with AL 8810 (10 μM) or depleted of the FP receptor using a targeting siRNA (siFP). Fra-1 upregulation by AA was completely blocked by both the FP antagonist and the siFP, demonstrating that AA-induced Fra-1 expression is mediated by PGF_2α_ via the FP receptor. (Fig. 5D, E).

To further investigate the signaling pathway activated by AA that upregulates Fra-1 expression, 3T3-L1 cells were pretreated either with a PKC inhibitor (GF109203X, 10 μM) or a MEK 1/2 inhibitor (U0126, 10 μM) prior to a 24 h AA treatment (100 μM). Both inhibitors significantly blocked the effect of AA on Fra-1 protein levels, suggesting a role for both PKC and ERK pathways in Fra-1 upregulation by AA (Fig. 5F, G).

### Short-term AA treatment of preadipocytes inhibits their differentiation potential via Fra-1

**Fig. 6.**
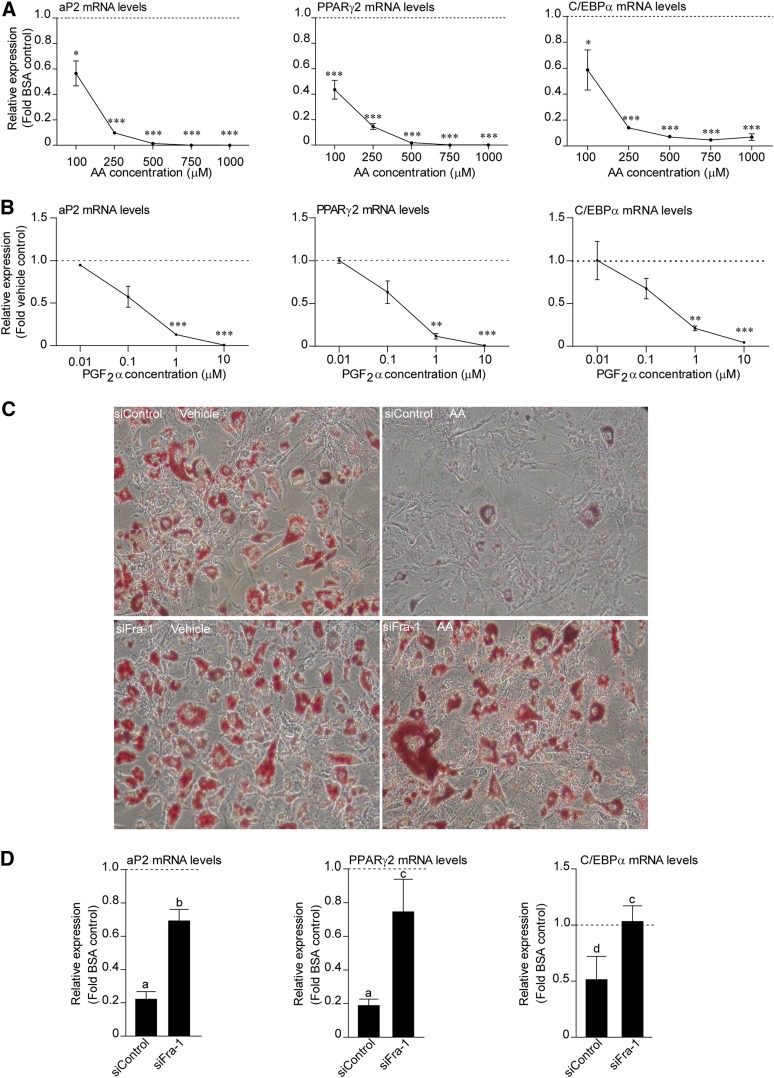
Fra-1 mediates the inhibition of adipocyte differentiation by AA. A: 3T3-L1 cells were treated with increasing doses of AA for 24 h in the presence of MDI. After the 24 h, AA was withdrawn and total RNA was isolated on day 10 of the differentiation and subjected to RT-PCR for aP2, PPARγ2, and C/EBPα. Data are presented as mean ± SEM based on triplicate determinations. *P < 0.05, **P < 0.01, ***P < 0.001. B: As above, but cells were treated with increasing doses of PGF_2α_. C: Eighty percent confluent 3T3-L1 cells were transfected with either siFra-1 or siControl. On the third day of transfection, cells were treated with 500 μM AA for 24 h in the presence of MDI. The next day, AA was withdrawn and the differentiation protocol continued until day 10. Cells were fixed and stained with Oil Red O and pictures were captured under a light microscope (20× magnification). D: Cells were transfected and treated as described in (C). Total RNA was isolated on day 10 and RT-PCR was performed. Data are presented as mean ± SEM of n = 3. A Student’s *t*-test was performed for BSA and AA-treated siControl (a: *P* < 0.001, d: *P* < 0.05) and for AA-treated siControl and siFra-1 (b: *P* < 0.001, c: *P* < 0.05).

We addressed whether the downstream role of AA-mediated changes in gene expression at the early stages of differentiation is to impact the subsequent differentiation potential of preadipocytes. For this, cells were treated with increasing doses of AA for the first 24 h of differentiation, then AA was withdrawn and the standard differentiation protocol was followed until day 10 when cells were harvested. The expression of three late differentiation markers, aP2, PPARγ2, and C/EBPα, at day 10 was much lower in the AA-treated cells compared with the vehicle control-treated cells for all doses tested (**Fig. 6A**). The inhibitory effect of AA was maximal when present early in the differentiation (during the first 2 days) in comparison to when it was present during later stages (after the first 2 days) (data not shown). It has to be noted that the aP2 expression was downregulated to an extent similar to the other differentiation markers tested, indicating that the effect of AA is on the differentiation program and not on the expression of specific genes (in contrast with the aP2-specific effect after 24 h of treatment, Fig. 1). Finally, treatment with PGF_2α_ for the first 24 h of differentiation could also inhibit differentiation (Fig. 6B).

Fra-1 expression was increased upon AA treatment at the first stages of differentiation (Fig. 5A). Thus, it was hypothesized that Fra-1 mediates the AA-dependent inhibition of differentiation. To test this, 3T3-L1 cells were transfected with siRNA targeting Fra-1 (siFra), then treated with AA (500 μM) for the first 24 h, and the effect on the differentiation was evaluated on day 10 either by Oil Red O stain or by RT-PCR for aP2, PPARγ2, and C/EBPα. Cells treated with AA and transfected with siControl showed greatly reduced lipid accumulation, whereas cells that were transfected with siFra-1 accumulated lipids at similar levels to the siControl vehicle-treated levels (Fig. 6C). In agreement with this observation, expression of all the differentiation markers tested in the presence of siFra-1 was similar to the vehicle-treated cells (Fig. 6D). These results confirmed the hypothesis that induction of Fra-1 levels upon AA treatment at the start of the differentiation procedure mediates the inhibition of differentiation by AA.

## DISCUSSION

The results in this study reveal a new mechanism by which AA regulates gene expression early in adipogenesis that ultimately directs terminal differentiation. We show that AA upregulates the expression of aP2 and Fra-1 in preadipocytes, two genes with important roles in adipogenesis. The first step of the signaling cascade is the metabolism of AA by COXs in preadipocytes. The expression of both COX isoforms is affected by the presence of AA (43), but also by agents included in the adipocyte differentiation cocktail (MDI) that elevate cAMP levels (11), suggesting possible synergistic pathways activated by AA and MDI regulating aP2 expression.

Several classes of prostanoids are synthesized in preadipocytes and PGs have been implicated in adipocyte differentiation having opposing effects. PGI_2_ and 15-deoxy-Δ^12,14^ PGJ_2_ promote (12–14), whereas PGF_2α_ and PGE_2_ inhibit adipocyte differentiation (16–18). We identified that PGF_2α_, over the other PGs produced, mediates the AA-activated increase in aP2 and Fra-1 expression in the early stages of adipogenesis. Our data and the fact that the levels of PGs are higher in preadipocytes compared with differentiated adipocytes (46), indicate that there is a window early in adipogenesis when preadipocytes are sensitive to specific stimuli and respond with the production of PGs.

PGF_2α_ signals via the FP receptor, a GPCR known to couple to Gαq/11. This receptor is linked with a variety of downstream signaling pathways, including the activation of different kinases. In this study, we identified that AA, via PGF_2α_ FP receptor activation, causes a transient calcium response and activation of PKC and ERK, which upregulate aP2 and Fra-1 expression. ERK signaling has been shown to be important for the early steps of the adipocyte differentiation program (47), but also for AA metabolism, as COX-2 is also regulated by the MEK/ERK pathway via the FP receptor (48). In addition, ERK is an important regulator of Fra-1 expression (49), activating its transcription (34, 50), stabilizing Fra-1 protein (51), and regulating Fra-1 transactivation activity (36). ERK signaling can regulate cellular differentiation depending on the strength, duration, and location of ERK activity (52), and in the context of adipogenesis, sustained ERK activation can inhibit differentiation (47). Our data suggest that the sustained ERK activation by AA in preadipocytes leads to the upregulation of aP2 and Fra-1 expression.

AA itself, but also 15-deoxy-Δ^12,14^ PGJ_2_, have been described to be direct ligands for PPARγ (13–15), making AA a good candidate for regulating adipogenesis. However, in vitro cell models yielded different results, showing AA to act both pro-adipogenically and anti-adipogenically (5–10). Animal feeding studies showed the same conflicting results (6, 7), and a similar scenario was also observed in studies of COX-2 (53, 54). However, it has been proposed that cAMP levels govern the action of AA on adipogenesis (10). Our results reveal that Fra-1 upregulation by AA in preadipocytes is the key event in the mechanism by which short-term exposure to AA can program the differentiation potential of preadipocytes. Thus, the diversity found in different cell and animal models could lie in the different Fra-1 expression levels likely to be governed by ERK activation, a strong Fra-1 regulator. In addition, our observations are in agreement with in vivo data, as depleting Fra-1 in 3T3-L1 cells does not perturb differentiation (this study) in accord with normal adipose tissue observed in Fra-1 knockout mice (55). A defect is apparent when Fra-1 is overexpressed, as it appears to act as a brake on the differentiation procedure, inasmuch as Fra-1 transgenic mice become lipodystrophic (30). However, the mechanism by which Fra-1 can cause the inhibition of differentiation still remains to be determined. It has been shown that PGF_2α,_ via ERK activation, can inhibit PPARγ activity (44), raising the possibility that Fra-1 is acting through PPARγ, as shown for Fra-2 (56). It is possible that early in differentiation, AA could modulate PPARγ activity by phosphorylation via the production of PGF_2α_, which in turn can decrease the transcriptional activity of PPARγ, inhibiting future maturation. In addition, new data suggest that aP2 can also inhibit adipocyte differentiation by downregulating PPARγ (57). Thus, our study suggests that upregulation of both aP2 and Fra-1, in response to AA, inhibits adipogenesis and highlights their increasingly evident importance in adipocyte differentiation (56, 57).

In conclusion, this is the first report showing that AA, through PGF_2α_ signaling, regulates aP2 and Fra-1 expression at the early stages of preadipocyte differentiation. We demonstrate that Fra-1 upregulation at the early stages causes the inhibition of adipocyte differentiation by AA, opening new possibilities to manage diet-induced obesity.

## Supplementary Material

Supplemental Data
