# Supplementary material for: Arachidonic acid-dependent gene regulation during preadipocyte differentiation controls adipocyte potential
Source: J Lipid Res. 2014 Dec;55(12):2479–90. doi: 10.1194/jlr.M049551 (PMC4242441; doi:10.1194/jlr.M049551)
Supplement: Supplemental Data [file supp_55_12_2479__index.html]

Arachidonic acid-dependent gene regulation during preadipocyte differentiation controls adipocyte potential — Arachidonic acid-dependent gene regulation during preadipocyte differentiation controls adipocyte potential — Supplemental Data 

# Arachidonic acid-dependent gene regulation during preadipocyte differentiation controls adipocyte potential

## Supplemental Data

**Files in this Data Supplement:**

- Supplementary figure 1 - Supplementary Figure 1: COXs expression is increased upon AA treatment in 3T3-L1 cells.
