# Supplementary material for: Arachidonic acid-dependent gene regulation during preadipocyte differentiation controls adipocyte potential
Source: J Lipid Res. 2014 Dec;55(12):2479–90. doi: 10.1194/jlr.M049551 (PMC4242441; doi:10.1194/jlr.M049551)
Supplement: Supplemental Data [file supp_M049551_jlr.M049551-1.pdf]

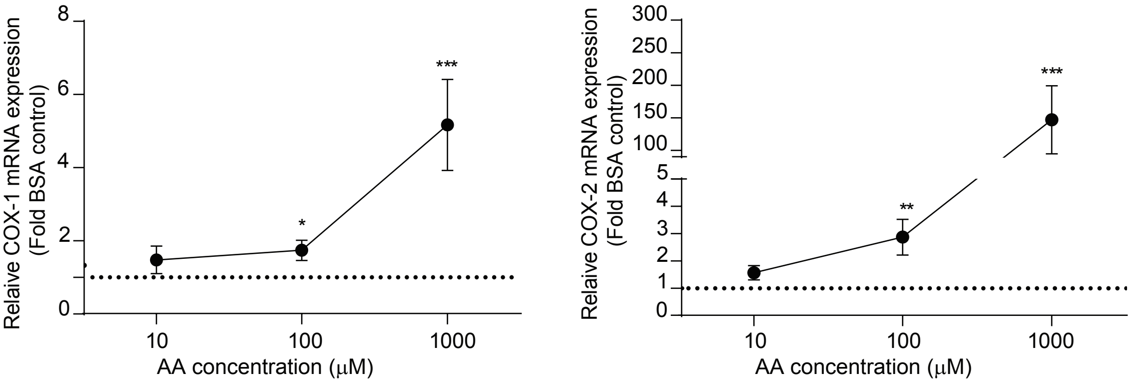

Supplementary Figure 1: COXs expression is increased upon AA treatment in 3T3-L1 cells. 3T3-L1 cells (D0) were treated with 10μM, 100μM or 1mM of AA for 24h in the presence of MDI. Total RNA was isolated and qRT-PCR was performed for COX-1 and COX-2 transcripts. Data are presented as mean±SEM of n=3.*P< 0.05, **P< 0.01, ***P<0.001.
